# Immune thrombocytopenic purpura and legionella infection: A case report highlighting the association

**DOI:** 10.1016/j.idcr.2022.e01439

**Published:** 2022-02-01

**Authors:** Aneeqa Javed, Muhammad Junaid Alvi, Syeda Sahra, Vincent DeChavez

**Affiliations:** a Staten Island University Hospital, Staten Island, NY, USA; b Mount Sinai Hospital, Manhattan, NY, USA

**Keywords:** AST, Aspartate aminotransferase, ALT, Alanine aminotransferase, ITP, Immune thrombocytopenic purpura, IVIG, Intravenous immunoglobulins, TTP, Thrombotic thrombocytopenia purpura, *Legionella*, Immune Thrombocytopenic Purpura, Thrombocytopenia

## Abstract

Immune thrombocytopenic purpura (ITP) can be acquired or secondary to other drugs, infections, or autoimmune disorders. *Legionella* is a known intracellular organism that causes Legionnaire's disease and affects the lungs. Presented is the first case showing a direct association between *Legionella* and ITP. Our patient was a 61-year-old female with a past medical history of asthma whose clinical presentation was consistent with pneumonia secondary to *Legionella*. Her hospital course was complicated by critical bleeding with severe thrombocytopenia. She responded to antibiotics, steroids, and intravenous immunoglobulins (IVIG). Our case suggests an association between ITP and *Legionella* and emphasizes its timely diagnosis for appropriate treatment.

## Background

Immune thrombocytopenic purpura (ITP) can be an acquired disorder or secondary to other drugs, infections, or autoimmune disorders [1]. The pathophysiology behind ITP includes complex B and T cells abnormalities leading to platelet destruction in the bloodstream and antibody-mediated damage to megakaryocytes, which leads to decreased production of platelets [2]. *Legionella* is a known intracellular organism that causes Legionnaire’s disease and affects the lungs. Presented is the first case showing an association between Legionella and ITP.

## Case presentation

A 61-year-old female with a past medical history of asthma presented to the emergency department with one week of fever, nonproductive cough, and shortness of breath. She also reported hematochezia and epistaxis with increasing frequency and severity over the past week leading to her presentation. On admission, she was afebrile (temperature 99.2F). Her blood pressure was 106/68 mm Hg, and she was tachycardic with a heart rate of 143 beats per minute, and SpO2 was 95% on room air. Admission labs indicated a white blood cell count of 20.69 K/uL, red blood cell count of 3.56 M/uL, hemoglobin 10.2 g/dL, a platelet count of 2 K/uL, activated thromboplastin 25.8 s, INR ratio of 1.36, plasma prothrombin time 15.60 s and a sodium serum 129 mmol/L. Haptoglobin was elevated to 282 mg/dL. She also had acute kidney injury with increased anion gap metabolic acidosis and mild transaminitis with aspartate aminotransferase (AST) 124 U/L and alanine aminotransferase (ALT) 77 U/L. She received a unit of platelets, after which her platelet count improved to 24 K/uL, but her hemoglobin dropped to 8.1 g/dL. A peripheral smear showed the presence of giant platelets and anisocytosis, red blood cells were microcytic.

**Fig. 1:**
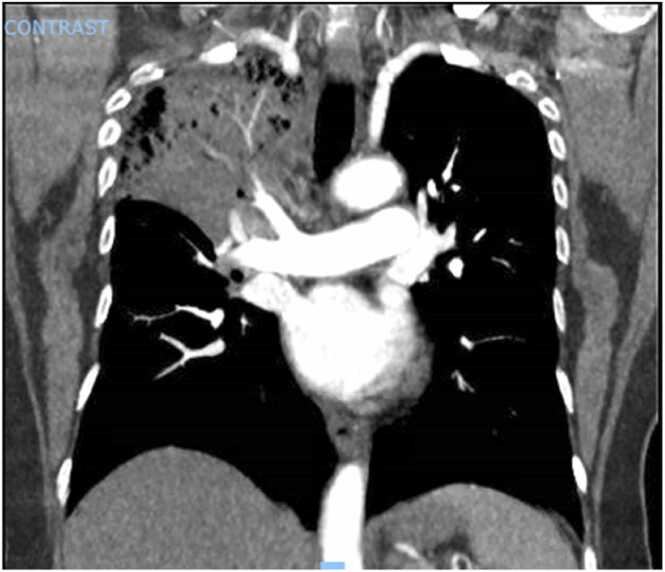
Opacification of the right upper lobe and patchy ground-glass opacities in the right middle, right lower, and left upper lobe.

**Fig. 2:**
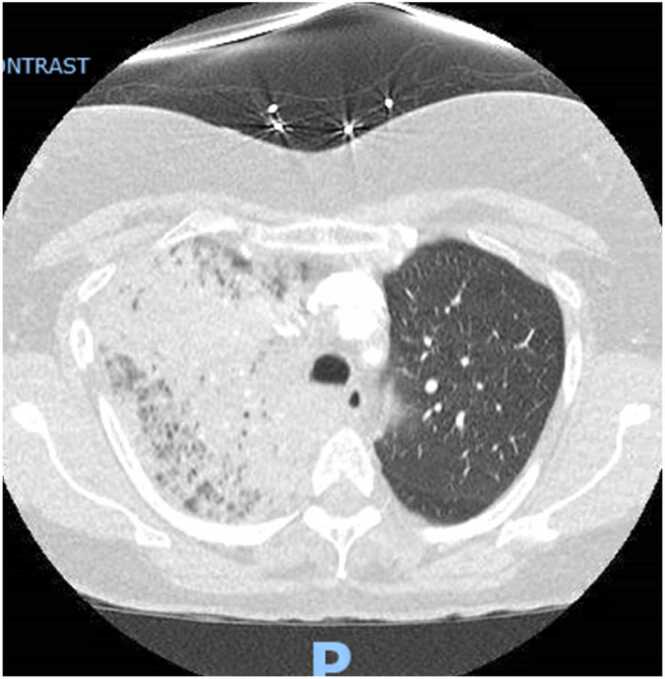
Opacification of the right upper lobe and patchy ground-glass opacities in the right middle, right lower, and left upper lobe.

Chest x-ray showed a consolidation in the right upper lobe of the lung, a subsequent computerized tomography (C.T.) scan of the chest showed near-complete opacification of the right upper lobe and patchy ground-glass opacities in the right middle, right lower, and left upper lobe (Figs. 1 and 2). Right upper lobe endobronchial airway filling defects were also seen. Other labs showed fibrinogen assay of more than 700 mg/dL, D-Dimer quantitative assay 947 ng/ml, lactate dehydrogenase 381 U/L, serum haptoglobin 282 mg/dL, and fibrinogen antigen 652 mg/dL. Total serum bilirubin never rose above 1 mg/dL. Direct bilirubin was not collected. Absolute reticulocytes count was 26 K/uL with reticulocyte percentage of 0.9. Peripheral smear was significant for decreased estimated platelet count. There was a slight microcytosis, polychromasia, and anisocytosis. She tested negative for hepatitis B and C and HIV. Urinalysis showed moderate blood. She was started on cefepime and levofloxacin for empiric coverage. She received three more platelets’ units and three units of packed red blood cells due to persistent thrombocytopenia, as low as 1 K/uL refractory to transfusions, suggesting autoimmune process and blood loss anemia.

**Fig. 3:**
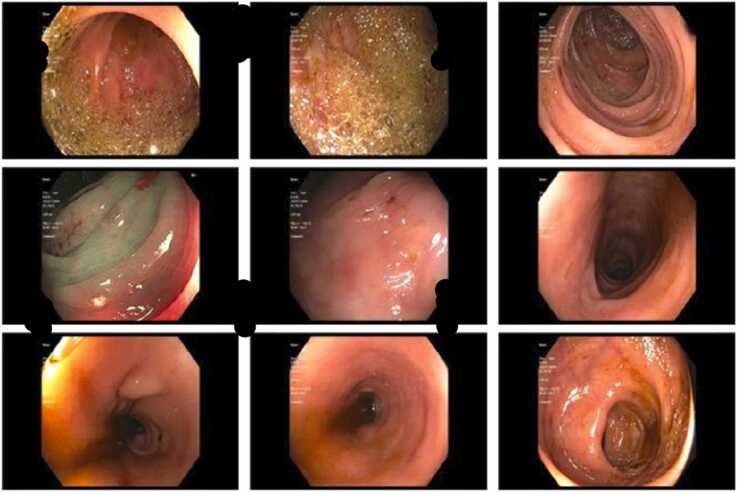
Internal and external hemorrhoids, polyp in the distal ascending colon, and erythema and petechiae in the whole colon.

**Fig. 4:**
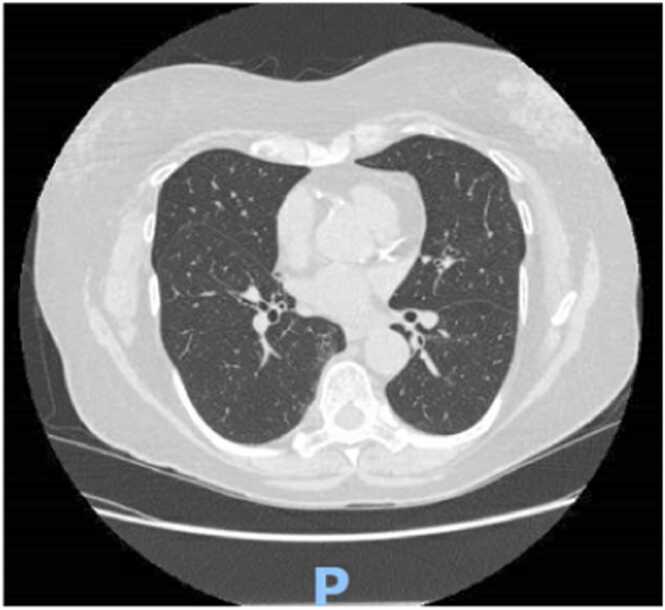
Improved multifocal pneumonia.

She received IVIG and high-dose steroids. The *Legionella* antigen in the urine, via immunochromatographic assay, was tested positive. The test used was BinaxNOW^R^ *Legionella* Urinary Antigen Card through concentrated urine samples. The test itself has 95% sensitivity and 95% specificity. The possibility of false positive or cross reactivity could not be ruled out. Antibiotics were narrowed to levofloxacin. Peripheral flow cytometry showed no blast population and normal myeloid granularity. Molecular testing was negative for JAK2 kinase mutation and the BCR-ABL gene. Anti-nuclear antibody (ANA) and Rheumatoid factor (RF) were negative. Within 24 h of beginning steroids and IVIG, her platelet counts improved. C.T. imaging revealed a normal-sized spleen. She underwent a colonoscopy during her stay and was found to have internal and external hemorrhoids, polyp in the distal ascending colon, and erythema and petechiae in the whole colon (Fig. 3); biopsies were taken, which showed no pathologic diagnosis. She received IVIG for three days, with improvement in her symptoms and hematologic lab values. She was discharged one week later with a platelet count of 293 K/uL and a hemoglobin of 8.8 g/dL. A month later, she underwent a repeat C.T. chest outpatient that showed overall improved multifocal pneumonia (Fig. 4). The prednisone was tapered down by hematology in the outpatient setting.

## Discussion and conclusion

ITP, previously idiopathic thrombocytopenic purpura, now stands for immune thrombocytopenic purpura. It can be an acquired primary disorder and a secondary disorder in the presence of infections, drugs, or other autoimmune disorders. The standard initial treatment for ITP is corticosteroids, and IVIG is recommended for patients with critical bleeding [3]. With regards to infection, inciting events leading to the development of ITP include Human Immunodeficiency Virus, hepatitis C virus, cytomegalovirus, varicella-zoster virus, and *Helicobacter pylori.*

*Legionella pneumophila* is an intracellular gram-negative bacterium known to cause Legionnaire's disease, a systemic infection primarily affecting the lungs, and Pontiac fever, a less severe flu-like illness [4].

To the best of our knowledge, only three published articles describe any association between Legionella pneumonia and ITP [5], [6], [7]. These articles have described possible mechanisms leading to thrombocytopenia, including complement activation, and inciting thrombotic thrombocytopenia purpura (TTP). *Legionella pneumophilia* can cause complement activation by binding to complement component C1q independently. Human platelets have C1q receptors, and interactions between C1q in an immune complex and C1q receptors cause platelet activation and aggregation resulting in platelet consumption and the development of microthrombi [7]. Additionally, in a case associated with TTP, the authors hypothesized that antibodies against *Legionella* cross-reacted with ADAMTS-13, an enzyme in which, in its deficient state, prevents von-Willebrand Factor from breaking down, in turn, promoting microthrombi and platelet consumption.

Our case is the first to show a direct association between Legionella and ITP. Our patient presented with critical bleeding due to severe thrombocytopenia and clinical findings consistent with *Legionella* pneumonia. Moreover, her thrombocytopenia could not be attributed to disseminated intravascular coagulation or thrombotic thrombocytopenic purpura. As ITP is a diagnosis of exclusion, this diagnosis is further supported by her response to steroids and IVIG.

*Legionella pneumophila* should always be considered in patients presenting with community-acquired pneumonia, and thrombocytopenia though rare is a crucial manifestation of *Legionella*. Our patient presented with critical bleeding due to severe thrombocytopenia and signs and symptoms of pneumonia. The causative organism was found to be *Legionella pneumophila*. She received steroids and IVIG, which improved her platelet count and antibiotics for her pneumonia. Timely diagnosis is necessary to ensure appropriate treatment.

## Ethical approval

The manuscript was reviewed and approved by Research Department and Ethics Committee.

## Funding

We obtained no funding from any organization or personnel during any stage of manuscript writing or submission.

## Consent

Written consent was obtained from patient to use personal and clinical details and images along with any identifying information, for the purpose of publishing this report.

## CRediT authorship contribution statement

The manuscript was written, and a literature review was obtained by A.J., J.A. S.S. and V.D., contributing to manuscript writing and revisions. All authors have read and approved the final manuscript.

## Conflict of interest statement

No competing financial or personal interests are involved for all the authors.

## Data Availability

The datasets used and analyzed during the current case reports are available from the corresponding author on reasonable request.
